# The Development and Validation of Job Satisfaction Questionnaire for Health Workforce

**DOI:** 10.21315/mjms2020.27.6.12

**Published:** 2020-12-29

**Authors:** Nurul Fatma Diyana Ahmad, Alex Kim Ren Jye, Zubalqiah Zulkifli, Mohamad Adam Bujang

**Affiliations:** 1 Medical Division, Sarawak State Health Department, Ministry of Health, Sarawak, Malaysia; 2 Quality Unit, Sarawak General Hospital, Sarawak, Malaysia; 3 Clinical Research Centre, Sarawak General Hospital, Sarawak, Malaysia

**Keywords:** health workforce, job satisfaction, questionnaire development, reliability, validity

## Abstract

**Background:**

This study aims to develop and validate a job satisfaction questionnaire (JS-Q) for health workforce who are employed by a healthcare institution.

**Methods:**

The study consists of six phases which begins with eliciting a conceptual understanding of the subject matter which is then followed by questions development, designing the overall structure and format of the questionnaire, assessing both its content validity and face validity, conducting a pilot study and finally a field test. A sample of study respondents who were permanent hospital staff above 18 years of age had been recruited from three government hospitals in Kuching, Sarawak, Malaysia.

**Results:**

The finalised JS-Q consists of a total of 34 questions that were based on 8 domains. For all these 8 domains, the minimum loading of each item on the factors was calculated to be at least 0.500, its coefficient of Cronbach’s alpha was calculated to be at least 0.750 and its corrected item-total correlation was calculated to be at least 0.500. The goodness of fit of the model was determined to be satisfactory with a value of Chi-square/df < 3.0, and a value of root mean square error approximation (RMSEA) < 0.8 and finally with both Tucker Lewis index (TLI) and comparative fit index (CFI) > 0.9.

**Conclusion:**

This newly developed and validated questionnaire (JS-Q) is found to be a valid and reliable study instrument for assessing job satisfaction among health workforce.

## Introduction

The healthcare industry increasingly requires a skilled workforce due to rapid advancements in medical technology, in concert with an ever-increasing expectation of patients towards more sophisticated methods for the optimal delivery of patient care. Job satisfaction among healthcare employees is essential for attracting and retaining top performers within the healthcare industry, by improving staff morale within the organisation. This is because job dissatisfaction can be a major cause of absenteeism and turnover among healthcare employees, which can adversely affect employees’ organisational commitment and the quality of healthcare services rendered (1–2).

Job dissatisfaction can be a major cause for concern within a knowledge-based sector such as the healthcare industry. In fact, such a heavy emphasis being placed on job satisfaction for the employees from the knowledge-based sectors (for example, the healthcare industry) shows that it is as important in these sectors as in the other business sectors. This is particularly true for the professional and service-based organisations such as hospitals, where the provision of long-term specialist training for medical professionals and retaining these highly skilled healthcare staff are deemed highly important (1).

This study aims to develop a new job satisfaction questionnaire (JS-Q) for health workforce. Although many existing varieties of JS-Q are already widely available in the literature, our intention for this study is to develop this questionnaire and to tailor its psychometric properties to be feasible for assessing job satisfaction among the entire local healthcare workers, in order to provide a mechanism for the continuous assessment and monitoring of their job satisfaction, especially within the local healthcare setting.

Job satisfaction serves an important function by enhancing the level of employee’s motivation and productivity (2–3). It is also an important measure which enables the top management and policymakers to constantly monitor the level of job achievement, so that they can seek various means to upgrade their job management and job enhancement. Without proper monitoring of job satisfaction, it is possible for employee behaviour to have a negative impact on their working environment and subsequently their productivity (4). Although the term ‘job satisfaction questionnaire’ is usually regarded as a common study instrument, it is however still necessary to revisit the specific domains and items for the measurement of job satisfaction, considering a myriad of challenges that are currently faced by these employees in their working environment nowadays, especially in the healthcare sector.

Hence, this newly developed questionnaire will prove very useful for providing continuous feedback to the top management as well as healthcare policymakers of the medical institutions on the level of job satisfaction reported by the health workforce from time to time. Such a feedback provided by the existing health workforce will immediately alert them about any adverse working conditions that present as factors which result in job dissatisfaction among these employees. This will hopefully prompt the authorities to identify the root cause of job dissatisfaction, in order to take all the necessary steps to address it by increasing their job satisfaction as well as making sure they are being heard with regard to their employment needs.

## Methods

### Population and Sample

In this study, the study population refers to all the healthcare employees who are currently working in Sarawak, Malaysia. A sample of study respondents was recruited from three government hospitals in Kuching, Sarawak which include Sarawak General Hospital, Bau Hospital and Lundu Hospital. All these study respondents recruited for this study were permanent hospital staff of above 18 years of age.

### Data Collection

The study respondents were recruited by using the snowball sampling technique, which involved the use of their email addresses. The researchers sent an email to each study participant which provided a link to the google form of the JS-Q for him/her to fill in.

### Process of Questionnaire Development

**Figure 1:**
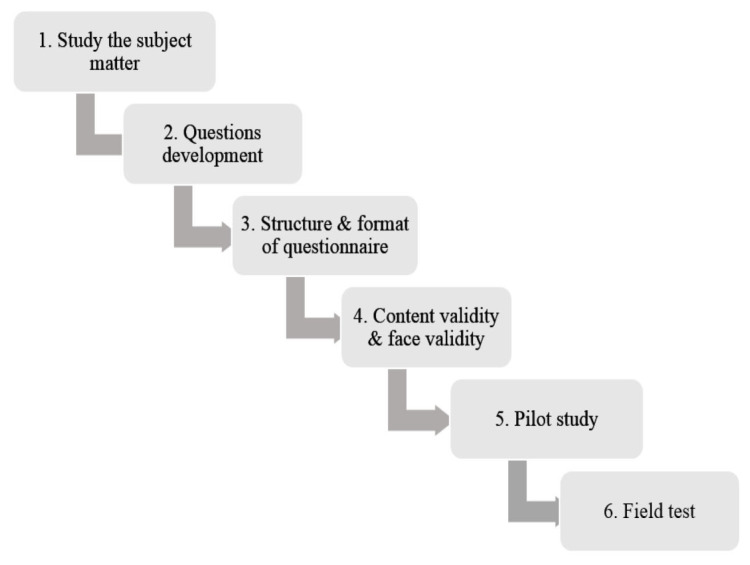
The recommended process for questionnaire development

The whole process of developing this questionnaire has been summarised in Figure 1. It involved a total of six phases starting from an initial exploration of the subject matter which encompassed the overall conceptualisation of the underpinning theory of job satisfaction. This was also an observational study that utilised both a qualitative approach (i.e. to study the background subject matter, to develop the specific items of the questionnaire, to determine both the structure and format of questionnaire and finally to assess its content & face validity) and a quantitative approach (i.e. to conduct a pilot study and a field test) to develop the scale for the measurement of job satisfaction.

**Figure 2:**
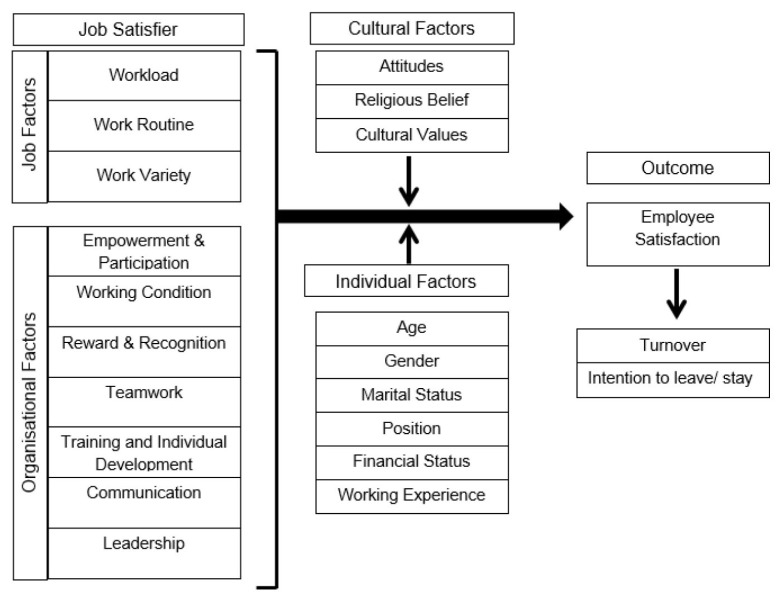
The conceptual framework for JS-Q

This research adopted the conventional framework for the development of measurement scales in which all the authors begin by taking the first step to conduct an extensive literature review of job satisfaction to enhance their understanding of the subject matter by exploration before developing the conceptual framework for this JS-Q, as shown in Figure 2 (1–9). Upon identification of a list of core components of the concept of ‘job satisfaction’, the authors then proceeded to develop the items of this JS-Q which were further categorised in 7 different domains. Hence, the first draft of the questionnaire was initially prepared, which included a total of 43 items within 7 domains namely i) ‘Empowerment and participation’ with 6 questions; ii) ‘Working condition’ with 8 questions; iii) ‘Reward and recognition’ with 6 questions; iv) ‘Teamwork’ with 5 questions; v) ‘Training and Development’ with 5 questions; vi) ‘Communication’ with 7 questions and vii) ‘Leadership’ with 6 questions.

Its content validity was assessed by three subject matter experts (SMEs) and this panel of SMEs included a high-ranking medical officer, an administrative officer providing diplomatic services who was holding a Master’s degree in human resource and a research consultant holding an MBA qualification. The final draft of the questionnaire was then pre-tested among 10 healthcare workers to determine its face validity. Its face validity was found to be satisfactory with only a few minor modifications necessary. Next, a total of 30 healthcare workers were then recruited for conducting a pilot test of this JS-Q, from which the reliability of this JS-Q was assessed to be satisfactory, as measured by its high value of internal consistency (Cronbach alpha > 0.800).

Finally, this questionnaire was field-tested within 1 month’s time from April 2019 until May 2019, before they were used in the main study. This field-testing of JS-Q involved conducting a cross-sectional survey among a group of study respondents who were permanent healthcare workers recruited from three hospitals in Kuching, Sarawak. In this survey, each study respondent was required to fill in a self-administered questionnaire that consists of questions on each individual’s socio-demographic profile and his/her job satisfaction level.

### Statistical Analysis

The ultimate aim of this study was to develop a new questionnaire that can accurately assess job satisfaction. Descriptive statistics were initially used to describe the socio-demographic profiles of all the study respondents. Exploratory factor analysis (EFA) and confirmatory factor analysis (CFA) were then conducted to determine the construct validity which then led to the selection of the best construct in this JS-Q for assessing the job satisfaction level. In order to verify the construct validity (which comprises both discriminant and convergent validity), the EFA was conducted by utilizing principle component analysis (PCA) with the varimax rotation method, which had applied an Eigenvalue of > 1 for this purpose. During this process, it is necessary to delete any items with either a cross loading of more than 0.40 or a loading of less than 0.40.

After using EFA to identify the factor structure present in a set of variables, the model fit was then assessed by using CFA where indicators such as Tucker Lewis Index (TLI ≥ 0.90), Comparative Fit Index (CFI ≥ 0.90), root mean square error approximation (RMSEA ≤ 0.08) and Akaike information criterion (AIC) were estimated (10–11). Deletion of items in the questionnaire was performed in a consecutive manner, and an item would be deleted if its content validity was too low and/or there were some abnormally large values associated with their error covariances from among the various items. This process of using CFA to test a hypothesised factor structure or model by assessing its goodness of fit to the data would continue until a satisfactory model fit has been achieved.

### Sample Size Planning

The determination of minimum required sample size was based on a rule-of-thumb for EFA since EFA has been applied to test the validity of the JS-Q. Initially, it was estimated that 40 items had been developed for the JS-Q. This means that based on the rule of thumb of using 5:1 ratio for sample size determination, the minimum sample size of 40 × 5 = 200 study participants would be required for using the EFA for this questionnaire development study (12–14).

## Results

### Baseline Sociodemographic Profile of the Respondents

A total of 343 study respondents were participating in this study, with a majority being female (74.8%) whose age ranges from 35 to 44 years (39.4%). More than three-quarters of these respondents are married (79.7%). Half of them are Bidayuh (50%). The largest proportion of these respondents are from the Medical Service Department (55.3%). Nearly half of them are nurses or community nurses (42.1%), whose job grades range from 27–40 (43.7%). The highest number of respondents consists of those who had worked in the hospital for more than 10 years (43.9%) whose monthly salary ranges from RM3,000 to less than RM5,000 (43.9%) (Table 1).

### Job Satisfaction Questionnaire

After conducting EFA using the PCA with varimax rotation method, which extracted those factors with Eigenvalues > 1, the proposed JS-Q questionnaire had been designed to consist of 39 questions that were categorised in 8 domains, namely: i) Leadership (8 questions); ii) Training and development (5 questions); iii) Teamwork (7 questions); iv) Empowerment and participation (5 questions); v) Working condition (3 questions) and vi) Reward and recognition (5 questions); vii) Communication (3 questions) and viii) Flexibility of working hours (3 questions). The domain of ‘Working condition’ was split into 2 sub-domains, namely: working condition and flexibility of working hours. All the items in the JS-Q were found to fit well within their respective domains, based on their content and also through determination by using relevant statistical computations. For all the domains of JS-Q, the minimum factor loading for each item was 0.500, the value of coefficient of Cronbach’s alpha was at least 0.850 and its corrected item-total correlation was at least 0.600.

The construct which was developed by using EFA was later re-examined using CFA to determine its model fit. The Chi-square/*df* was 2.739 with RMSEA was 0.067 although both TLI and CFI were slightly lower than 0.90 (Table 2). Five items were deleted because they did not demonstrate adequate content validity (since they failed to result in measures that will adequately sample the theoretical domain of interest) and also there was an excessive amount of covariance error terms (as suggested by the modification indices of the measurement model). These five items were deleted one by one until the model reached a satisfactory model fit.

The final version of the JS-Q now consists of only 34 items that were categorised in 8 domains, namely: i) Leadership (5 questions); ii) Training and development (5 questions); iii) Teamwork (5 questions); iv) Empowerment and participation (5 questions); v) Working conditions (3 questions); vi) Reward and recognition (5 questions); vii) Communication (3 questions) and viii) Flexibility of working hours (3 questions). Several indicators of the goodness of fit of the model were found to be satisfactory with Chi-square/*df* < 3.0, RMSEA = 0.06, both TLI and CFI were higher than 0.9 and also its AIC = 1467.8 was found lower than that of the original version of JS-Q which consists of 39 questions (AIC = 2151.1) (Table 2).

Next, the construct of the final version of JS-Q was tested by using EFA which revealed that the EFA factor solution did not adequately incorporate all the total 8 domains into the conceptual framework of JS-Q. However, when EFA was conducted on the same subset of participants for determining the factor structure of the 34-items of the final version of JS-Q, and such factor analyses were repeated until a solution in which all the items included in the analysis had met all these criteria was obtained, it was again found that the same 8 domains together with their respective items would then be incorporated into the conceptual framework of JS-Q after the EFA had successfully extracted an 8-factor solution in a 34-item measure. For all these 8 domains extracted by EFA, the minimum factor loading for each item was 0.500, the value of coefficient of Cronbach’s alpha was at least 0.750 and its corrected item-total correlation was at least 0.500 (Table 3).

## Discussion

The JS-Q was initially developed and validated as a new, self-administered instrument for measuring job satisfaction among all the hospital staff. The final construct of the JS-Q has now been designed to consist of a total of 8 domains with 34 items, along with an additional domain (namely, flexibility of working hours) being incorporated into the original conceptual framework of the study (Figure 2). Thus, the conceptual framework of this JS-Q is now based on 8 domains, namely: i) Leadership; ii) Training and development; iii) Teamwork; iv) Empowerment and participation; v) Reward and recognition; vi) Communication; vii) Working conditions and viii) Flexibility of working hours.

It is likely for both leadership style and organisational culture to have a positive influence on an employee’s level of job satisfaction, especially when the leaders have a vision which is aligned with their organisational culture, which typically occurs within the framework of a transformational leadership. As a result, both employees and their superiors will cooperate not only for the sake of organisation’s well-being, but also for the fulfilment of their personal needs and desires (7). Meanwhile, the provision of in-house training for human resource development will provide numerous benefits to an organisation, especially in relation to preventing errors, improving workplace safety and decreasing staff turnover. It may also be helpful for an organisation to cultivate a learning environment, which may promote innovation and impart a friendlier and more conducive organisational culture. To do so, it is necessary to specifically allocate adequate financial resources for expanding such efforts (9).

Executive leaders and managers should reinforce the importance of teamwork among all employees because effective teamwork will enable them to align themselves towards a common goal, thereby enhancing employees’ motivation and a sense of belonging, which will indirectly boost their level of job satisfaction. By doing so, the employees will also be motivated to accomplish a mutual goal (7). On the other hand, the empowerment of employees and their participation should first be initiated by the top management. To do so, it involves moving the capacity for decision-making to the lowest possible level within the organisation. Hence, the employers will empower all the employees by strongly encouraging them to open up by meeting together to discuss any matters arising from their work, as well as any other matters arising from their job functions, whether it involves managerial decisions and/or high-level policies (7). Besides that, organisations will also have to develop a formal reward and recognition system to enlist the support of employees, by engaging them in teamwork. In order to ensure that due recognition will be given to any significant contribution and/or effort made by an employee, all the departments of an organisation should also be rewarded as a gesture to promote and support the attainment of a particular performance level as a common goal within the whole organisation (8).

Communication is a basic tool that is used at all levels of the working environment as well as between the top management and the workforce at the ground level. Managers will be better equipped to foster job satisfaction and effective organisational commitment through a proper channel for internal communication wherein they recognise and acknowledge all the input from the employees. Managers must have a clear understanding of both the quantity and quality of information sought by the employees if they intend to design an effective two-way internal communication channel that meets the information needs of an employee (5). By doing so, all the employees will regularly be provided with information from the management from time to time, and are also given an opportunity to be heard by the management; thereby both the employees and the management staff will have a closer knit of working relationship together.

The initial items for specifying the concept of ‘working condition’ in JS-Q was split into two, namely ‘working condition’ and ‘flexibility of working hours’. The original conceptual framework of the construct of JS-Q had defined the term ‘working condition’ as ‘the condition under which a job has been performed’. This can be influenced by i) external factors that include climate; ii) subjective factors that include fatigue, monotony and unfavourable posture etc; iii) factors related to the organisation of production such as duration of work shift, design of work schedule and duration of working time, etc. (6).

It is commonly known that the working condition can have a significant effect on job satisfaction because it can influence the quality of the physical environment where they would be working. Therefore, the term ‘working condition’ can include many aspects of the working environment such as adequate workspace, appropriate level of lighting, minimal noise level, thermal comfort working area, the provision of essential utilities such as electricity and water supply, and the availability of office equipment. Management should also provide ergonomically-designed workspaces that enhance employees’ health and well-being (4). Flexibility of working hours is now becoming increasingly important among the workers. Many organisations now begin to offer flexible working hours to employees due to the potential benefits that such a flexibility can bring to both the employers and their employees. Examples of these benefits include a higher level of employee productivity along with a higher level of organisational profitability. Most importantly, the option of flexible working hours can also promote and maintain work-life balance among the workforce, which is an important aspect of a healthy work environment (15).

The existing literature on the many different facets of ‘job satisfaction’ have all supported the construct of all the expected 8 domains of a job satisfaction measure in JS-Q. In addition, the evidence of reliability and validity of the scales in JS-Q was also provided by both EFA and CFA. Although the re-analysis of the final construct of JS-Q by using the EFA with the added criterion of having an eigenvalue of > 1 for retaining the items for an ideal construct (in order to improve the overall model fit) did not manage to incorporate all the expected 8 distinct domains into the conceptual framework of the construct of this questionnaire, however we can nevertheless still be confidently using EFA to successfully extract an 8-factor solution in a 34-item measure by incorporating all the 8 domains together with each of their respective items into the conceptual framework of the construct of this JS-Q. This was done by conducting EFA on the same subset of participants to determine the factor structure of this questionnaire and these factor analyses were repeated until a factor solution which incorporated all the items that had fulfilled the criteria was identified. This justifies why the authors should rightfully insist on maintaining the existing 8 domains since they have already provided a good reflection of its conceptual framework which had been developed previously. Most importantly, all the model fit indices of the final construct of this JS-Q (consisting of 34 items) were found to be satisfactory, which means that the final construct of the questionnaire has a better model fit than the previous construct of JS-Q (consisting of 39 items).

This also explains why if the researchers conducted the EFA by using the criterion of having an eigenvalue of > 1 for retaining the items for an ideal construct, but did not manage to identify a construct consisting of 8 domains in the final construct of 34-item measure of JS-Q; then it will be strongly recommended for the researchers to conduct EFA again on the same subset of participants to determine the factor structure of this questionnaire, and then to repeat all the factor analyses until a factor solution in which all the items that have fulfilled all criteria are now included in the analysis. This is an important and necessary step for the researchers to identify a model specification that forces all the 8 domains into the factor solution during the factor analysis. In addition, as long as the internal consistency for each domain of the construct is found to be satisfactorily high (i.e. > 0.65) and the model fit was assessed to be satisfactory by CFA, then the construct of JS-Q can be considered a reliable and valid study instrument for measuring job satisfaction.

A major strength of this study is that its findings are demonstrated to have relevance in many important practical and theoretical applications. Apart from its use for measuring job satisfaction among the internal workforce within the organisation and also for research purposes, this study has identified a new independent domain which can be used to assess the job satisfaction dimension, namely flexible working hours. Moreover, this study has also determined the high significance of this new domain which is then subsequently incorporated it into the final construct of the JS-Q.

Another major achievement of this study for the validation of this JS-Q is that both its reliability and validity were found to be satisfactory, which was substantiated by the appropriate selection of the items for each of the respective domains together with an adequate sample size of study respondents who were recruited for this study. This study had recruited a total of 343 respondents and hence it is clear that this sample size has greatly exceeded the minimum sample size required for both EFA and CFA (12–14).

However, an important limitation of this study is that the authors of this study are well aware of the fact that although this JS-Q has been validated among healthcare workers, its psychometric properties (i.e. assessment of its validity and reliability) remain untested in workers of other fields. We therefore encourage the JS-Q to be validated in different languages as well as to apply it within various other types of organisations. For example, it is possible to replace the term ‘hospital’ with another term such as ‘healthcare organisation’ or ‘healthcare department’ so that the JS-Q can then potentially be applied in other types of organisational settings.

Insofar that it is the authors’ intention to generalise the applicability of this JS-Q to a broader range of study respondents, however it must still be noted that further studies are required to confirm its reliability and validity as a research instrument for use in workers of other fields since this study had recruited only healthcare workers as its study respondents. Therefore, it may not be valid for the results obtained from this study to be extrapolated to other study settings and/or study respondents apart from the healthcare industry.

## Conclusion

The JS-Q is found to be both a valid and reliable study instrument for measuring job satisfaction among the healthcare workers and can also be recommended for use in several other related purposes, such as in management and research that involve an assessment of job satisfaction. Hence, it is now recommended for the JS-Q to be used for measuring job satisfaction among healthcare workers. Lastly, the authors also strongly recommend the readers to apply the items contained in the JS-Q 34 items (Appendix 1) for use as a research instrument to measure job satisfaction among employees in many different organisational settings other than the healthcare sector, in which it had originally been developed and validated, as long as its psychometric properties have been fully tested and it has been found to be both a valid and reliable instrument prior to using it in all these settings.

## Figures and Tables

**Table 1:** Characteristics of respondents from government healthcare workers in Kuching division

	*n*	%
**Gender**		
Male	258	25.2
Female	87	74.8
**Age**		
21 to 34	108	31.5
35 to 44	135	39.4
45 to 54	75	21.9
55 or older	25	7.3
**Marital status**		
Divorced/Widowed	15	4.4
Married	271	79.7
Single	54	15.9
**Ethnicity**		
Bidayuh	169	50.0
Chinese	56	16.6
Iban	30	8.9
Malay	67	19.8
Melanau	9	2.7
Others	7	2.1
**Department**		
Administration	47	13.8
Diagnostic and clinical support	48	14.1
Medical service	188	55.3
Surgical service	24	7.1
Woman and child service	33	9.7
**Position**		
Administration staff	31	9.6
Allied health	36	11.2
Assistant medical officer	26	8.1
Medical care assistant	18	5.5
Medical officer	35	10.8
Nurse/Community nurse	136	42.1
Pharmacist/Assistant pharmacist	20	6.2
Specialist/Consultant	9	2.8
Others	12	3.7
**Work experience**		
Less than 1 year	18	5.3
1 year to less than 2 years	17	4.9
2 years to less then 5 years	60	17.4
5 years to less than 10 years	97	28.4
10 years or more	150	43.9

**Table 2:** Model fit indices of JS-Q 39 items versus JS-Q 34 items

Model fit indices	JS-Q 39 items	JS-Q 34 items
CMIN	2.739	2.404
Tucker Lewis Index (TLI)	0.884	0.0913
Comparative Fit Index (CFI)	0.891	0.919
RMSEA	0.067	0.06
AIC	2151.147	1467.766

**Table 3:** Result of EFA and internal consistency for JS-Q which consists of 34 items and 8 domains

Items	Domains in JS-Q								Cronbach’s alpha (min CITC)
	TW	LD	RR	EP	TD	WH	C	WC	
TW1	0.844								0.924 (0.781)
TW2	0.797								
TW3	0.787								
TW4	0.785								
TW5	0.699								
LD1		0.799							0.930 (0.755)
LD2		0.778							
LD3		0.758							
LD4		0.741							
LD5		0.628							
RR1			0.870						0.879 (0.720)
RR2			0.789						
RR3			0.771						
RR4			0.766						
RR5			0.607						
EP1				0.827					0.876 (0.608)
EP2				0.821					
EP3				0.808					
EP4				0.673					
EP5				0.529					
TD1					0.783				0.878 (0.716)
TD2					0.747				
TD3					0.693				
TD4					0.679				
TD5					0.600				
WH1						0.840			0.826 (0.590)
WH2						0.789			
WH3						0.592			
C1							0.735		0.871 (0.725)
C2							0.719		
C3							0.656		
WC1								0.795	0.751 (0.548)
WC2								0.740	
WC3								0.525	

Notes: Model fit indices based on CFA are described in Table 1; EFA was conducted based on PCA using the varimax rotation method and and the factor solution is forced into eight domains; TW = Teamwork; LD = Leadership; RR = Rewards and recognitions; EP = Empowerment; TD = Training and development; WH = Flexibility of working hours; C = Communication; WC = Working condition

## Appendix 1

### HOSPITAL INTERNAL CLIENT SATISFACTION SURVEY/*KAJI SELIDIK TAHAP KEPUASAN KAKITANGAN HOSPITAL*

Please read each statement carefully and determine the degree to which you agree with these statements below. Kindly take your organisation into consideration as you respond to each statement. Please mark only 1 answer for each statement.

*Sila baca setiap penyataan di bawah dengan teliti dan tentukan tahap sejauh mana anda bersetuju terhadap penyataan berikut. Sila pertimbangkan organisasi anda dalam menjawab setiap penyataan. Sila tanda hanya 1 jawapan bagi setiap penyataan*.

#### A. Teamwork (TW) / *Kerja Berpasukan*

**Table:**

	Strongly disagree/*Sangat tidak setuju*	Disagree/*Tidak setuju*	Uncertain/*Tidak pasti*	Agree/*Setuju*	Strongly agree/*Sangat setuju*
1. My co-workers are committed to doing quality work. *Rakan sekerja saya komited untuk melakukan kerja yang berkualiti*.	1	2	3	4	5
2. It is easy to get along with my colleagues. *Adalah sangat mudah untuk bergaul dengan rakan sekerja saya*.	1	2	3	4	5
3. I feel part of a team in working towards shared goals. *Saya merasakan saya berada di dalam satu pasukan dalam mencapai matlamat kerja yang sama*.	1	2	3	4	5
4. I experience a spirit of cooperation in this organisation. *Saya dapat merasakan semangat bekerjasama di dalam organisasi ini*.	1	2	3	4	5
5. I receive assistance from co-workers when necessary. *Saya mendapat bantuan dari rakan sekerja bila diperlukan*.	1	2	3	4	5

#### B. Leadership (LD) / *Kepimpinan*

**Table:**

	Strongly disagree/*Sangat tidak setuju*	Disagree/*Tidak setuju*	Uncertain/*Tidak pasti*	Agree/*Setuju*	Strongly agree/*Sangat setuju*
1. My supervisor/senior manager visibly demonstrates a commitment to quality. *Penyelia saya menunjukkan komitmen dalam menghasilkan kerja yang berkualiti*.	1	2	3	4	5
2. It is clear to me what my supervisor expects of me regarding my job performance. *Penyelia saya menerangkan dengan jelas mengenai harapan beliau berkenaan prestasi kerja saya*.	1	2	3	4	5
3. My supervisor is able to address my questions or concerns. *Penyelia saya dapat menangani soalan atau kebimbangan saya*.	1	2	3	4	5
4. My supervisor has strong management skills. *Penyelia saya mempunyai kemahiran pengurusan yang bagus*.	1	2	3	4	5

#### C. Reward and Recognition (RR) / *Ganjaran dan Pengiktirafan*

**Table:**

	Strongly disagree/*Sangat tidak setuju*	Disagree/*Tidak setuju*	Uncertain/*Tidak pasti*	Agree/*Setuju*	Strongly agree/*Sangat setuju*
1. My base pay is fair for my responsibilities. *Gaji pokok saya adalah berpatutan untuk pekerjaan saya*.	1	2	3	4	5
2. The annual raise is reasonable. *Kadar kenaikan gaji tahunan saya adalah munasabah*.	1	2	3	4	5
3. I am satisfied with the retirement plan. *Saya berpuas hati dengan pelan persaraan saya*.	1	2	3	4	5
4. The process used to determine promotions/annual raises is just and fair. *Proses kenaikan gaji tahunan dan kenaikan pangkat adalah adil dan saksama*.	1	2	3	4	5
5. I received the right amount of recognition or praise for work that is well done. *Saya menerima penghargaan yang sewajarnya untuk pencapaian cemerlang saya*.	1	2	3	4	5

#### D. Empowerment and Participation (EP) / *Penguasaan dan Penyertaan dalam Organisasi*

**Table:**

	Strongly disagree/*Sangat tidak setuju*	Disagree/*Tidak setuju*	Uncertain/*Tidak pasti*	Agree/*Setuju*	Strongly agree/*Sangat setuju*
1. I understand the vision of my organisation. *Saya memahami visi organisasi saya*.	1	2	3	4	5
2. The mission and purpose of my organisation make me feel that my job is important. *Misi dan matlamat organisasi membuatkan saya merasakan pekerjaan saya sangat penting*.	1	2	3	4	5
3. I feel I’ve contributed to the organisation’s plan and mission. *Saya merasakan saya menyumbang kepada rancangan dan misi organisasi*.	1	2	3	4	5
4. My job makes good use of my skills and abilities. *Kerja saya memanfaatkan kemahiran dan kebolehan saya*.	1	2	3	4	5
5. I am satisfied with my involvement in decisions that affect my work. *Saya berpuas hati dengan penglibatan saya dalam membuat keputusan di dalam pekerjaan saya*.	1	2	3	4	5

#### E. Training and Individual Development (TD) / *Latihan dan Perkembangan Individu*

**Table:**

	Strongly disagree/*Sangat tidak setuju*	Disagree/*Tidak setuju*	Uncertain/*Tidak pasti*	Agree/*Setuju*	Strongly agree/*Sangat setuju*
1. My initial training provided by the hospital was sufficient. *Latihan/kursus awal yang diberikan oleh pihak hospital kepada saya adalah mencukupi*.	1	2	3	4	5
2. As much ongoing training I need is provided by the Hospital. *Pihak hospital menyediakan latihan/kursus berterusan yang diperlukan oleh saya*.	1	2	3	4	5
3. Training offered by the organisation helps me to be effective and efficient in my job. *Latihan/kursus yang ditawarkan membantu saya menjadi lebih cemerlang dan cekap dalam kerja*.	1	2	3	4	5
4. The organisation encourages continued education and professional growth. *Organisasi ini menggalakkan pembelajaran berterusan dan perkembangan dalam kerjaya*.	1	2	3	4	5
5. I have opportunities at work to learn and grow. *Saya diberi peluang di tempat kerja untuk belajar dan mengembangkan kerjaya saya*.	1	2	3	4	5

#### F. Working Hours (WH) / *Masa Bekerja*

**Table:**

	Strongly disagree/*Sangat tidak setuju*	Disagree/*Tidak setuju*	Uncertain/*Tidak pasti*	Agree/*Setuju*	Strongly agree/*Sangat setuju*
1. I am satisfied with my total working hours. *Saya berpuas hati dengan jumlah waktu bekerja saya*.	1	2	3	4	5
2. I am given the flexibility in scheduling my working hours. *Saya diberikan kelonggaran dalam mengatur waktu bekerja saya*.	1	2	3	4	5
3. I have the flexibility to manage my work and nonwork interests e.g. caring responsibilities, study, sports interests, etc. *Saya diberi kelonggaran dalam menguruskan perkara yang berkaitan ataupun tidak berkaitan dengan pekerjaan saya seperti sukan, pembelajaran dan tanggungjawab lain*.	1	2	3	4	5

#### G. Communication (C) / *Komunikasi*

**Table:**

	Strongly disagree/*Sangat tidak setuju*	Disagree/*Tidak setuju*	Uncertain/*Tidak pasti*	Agree/*Setuju*	Strongly agree/*Sangat setuju*
1. This organisation does an excellent job of keeping employees informed about matters affecting us. *Organisasi ini telah berjaya menyampaikan informasi yang tepat berkenaan sesuatu perkara*.	1	2	3	4	5
2. Management clearly explains the reasons behind decisions on key issues. *Pihak pengurusan menyatakan sebab yang jelas di sebalik setiap keputusan berkaitan isu-isu utama*.	1	2	3	4	5
3. I know where to go within the organisation to obtain information that I need. *Saya mengetahui tempat rujukan di dalam organisasi saya untuk mendapatkan maklumat yang saya perlukan*.	1	2	3	4	5
4. My supervisor clearly communicates his/her expectations of my job performance. *Penyelia saya menyampaikan harapannya tentang prestasi kerja saya dengan jelas*.	1	2	3	4	5

#### H. Working Condition (WC) / *Persekitaran Kerja*

**Table:**

	Strongly disagree/*Sangat tidak setuju*	Disagree/*Tidak setuju*	Uncertain/*Tidak pasti*	Agree/*Setuju*	Strongly agree/*Sangat setuju*
1. My physical working conditions (lighting, ventilation, ergonomic, etc) are good. *Persekitaran kerja saya (pencahayaan, pengudaraan, ergonomik, dan lain-lain) sangat bagus*.	1	2	3	4	5
2. I have the tools and resources that I need in order to do my work right. *Saya mempunyai sumber dan alatan yang mencukupi untuk melakukan pekerjaan saya*.	1	2	3	4	5
3. The amount of work expected of me is reasonable. *Jumlah beban kerja saya adalah berpatutan*.	1	2	3	4	5

## References

[1] Ali-Mohammed MR, Ailson DM (2009). Factors affecting employees’ job satisfaction in public hospitals: implications for recruitment and retention. Journal of General Management.

[2] Ali-Mohammad MR, Ewan F, Duska R (2008). A study of the relationship between job satisfaction, organisational commitment and turnover intention among hospital employees. Health Serv Manage Res.

[3] Fengfan Z, Zhenni L, Ting C, Rui M, Pengqian F (2017). Factors affecting turnover intentions among public hospital doctors in a middle city in central China. Australian Health Review.

[4] Roelofsen C (2002). The impact of office environments on employee performance: The design of the workplace as a strategy for productivity enhancement. Journal of Facilities Management.

[5] Carrière J, Bourque C (2009). The effects of organizational communication on job satisfaction and organizational commitment in a land ambulance service and the mediating role of communication satisfaction. CAREER DEV INT.

[6] Bakotić D, Babić T (2013). Relationship between working conditions and job satisfaction: the case of Croatian shipbuilding company. International Journal of Business and Social Science.

[7] Belias D, Koustelios A (2014). Organizational culture and job satisfaction: a review. International Review of Management and Marketing.

[8] Saleem MA (2014). Relationship between job satisfaction and job performance: a case study of Universities of Peshawar District (KPK) Pakistan. European Journal of Business and Management.

[9] Asfaw AM, Argaw MD, Bayissa L (2015). The impact of training and development on employee performance and effectiveness: a case study of District Five Administration Office, Bole Sub-City, Addis Ababa, Ethiopia. Journal of Human Resource and Sustainability Studies.

[10] Bagozzi RP, Yi Y (1988). On the evaluation of structure equations models. J Acad Market Sci.

[11] Browne MW, Cudeck R, Bollen K, Long JS (1993). Alternative ways of assessing model fit. Testing structural equation models.

[12] Bujang MA, Ab-Ghani P, Soelar SA, Zulkifli NA (2012). Sample size guideline for exploratory factor analysis when using small sample: taking into considerations of different measurement scales.

[13] Bujang MA, Ghani PA, Soelar SA, Zulkifli NA, Omar ED (2019). Invalid skewed responses contributes to invalid factor solution in exploratory factor analysis: a validation approach using real-life data. J Behav Health.

[14] Wolf EJ, Harrington KM, Clark SL, Miller MW (2013). Sample size requirements for structural equation models: an evaluation of power, bias, and solution propriety. Educational and Psychological Measurement.

[15] Shagvaliyeva S, Yazdanifard R (2014). Impact of flexible working hours on work-life balance. American Journal of Industrial and Business Management.

